# H9N2 influenza viruses from Bangladesh: Transmission in chicken and New World quail

**DOI:** 10.1111/irv.12589

**Published:** 2018-09-08

**Authors:** Patrick Seiler, Lisa Kercher, Mohammed M. Feeroz, Karthik Shanmuganatham, Lisa Jones‐Engel, Jasmine Turner, David Walker, S. M. Rabiul Alam, M. Kamrul Hasan, Sharmin Akhtar, Pamela McKenzie, John Franks, Scott Krauss, Richard J. Webby, Robert G. Webster

**Affiliations:** ^1^ Department of Infectious Diseases St. Jude Children's Research Hospital Memphis Tennessee; ^2^ Department of Zoology Jahangirnagar University Dhaka Bangladesh; ^3^ Diagnostic Virology Laboratory National Veterinary Services Laboratories United States Department of Agriculture Animal and Plant Health Inspection Service Ames Iowa; ^4^ National Primate Research Center University of Washington Seattle Washington

**Keywords:** Bangladesh, chicken, disease, H9N2 influenza, quail, transmission

## Abstract

The H9N2 influenza viruses that have become established in Bangladeshi live poultry markets possess five gene segments of the highly pathogenic H7N3 avian influenza virus. We assessed the replication, transmission, and disease potential of three H9N2 viruses in chickens and New World quail. Each virus replicated to high titers and transmitted by the airborne route to contacts in both species. Infected chickens showed no disease signs, and the viruses differed in their disease potential in New World quail. New World quail were more susceptible than chickens to H9N2 viruses and shed virus after airborne transmission for 10 days. Consequently, New World quail are a potential threat in the maintenance and spread of influenza virus in live poultry markets.

## 1 INTRODUCTION

Eurasian H9N2 influenza viruses came to prominence in the mid‐1990s when they were shown to contribute the six internal gene segments of the highly pathogenic H5N1 influenza virus.^1^ Phylogenetically, the Eurasian H9N2 influenza viruses can be classified into three groups represented by prototype viruses, namely A/Quail/Hong Kong/97 (G1 lineage), A/Duck/Hong Kong/Y280 (Y280 lineage), and A/Chicken/Korea 38349—p96323/96 (Korean lineage).^2^ H9N2 viruses of the G1 lineage have spread throughout the poultry industry of Eurasia and are particularly prevalent in chickens, in which they cause low‐pathogenic inapparent infections unless the birds are coinfected with other poultry disease agents.^3^ During its spread in Pakistan, the G1‐lineage H9N2 influenza virus has reassorted with the highly pathogenic avian influenza (HPAI) H7N3 viruses and acquired their PB2, PB1, PA, and NS gene segments.^4^ These reassortant viruses cause severe infections in chickens and sporadic deaths in quail. In Bangladesh, the circulating H9N2 influenza virus arose from a different reassortment and possesses five gene segments (PB1, PA, NP, M, and NS) derived from HPAI H7N3 viruses.^5^ We have determined the replication, transmissibility, and disease potential of these Bangladeshi H9N2 viruses in chickens and New World quail.

The three specific viruses used in this study were A/environment/Bangladesh/10306/2011 (H9N2) (isolated from a quail cage, GenBank KC757959), A/chicken/Bangladesh/10450/2011 (H9N2) (GenBank KC757840), and A/quail/Bangladesh/19462/2013 (H9N2) (GenBank KJ643700). The viruses were propagated and titrated in the allantoic cavities of 10‐day‐old embryonated chicken eggs at 35°C for 48 hours as described previously.^5^ The birds used in the study were northern bobwhite quail (*Colinus virginianus*) aged 5‐6 weeks for Old World quail were not available to us. Previous studies have shown that bobwhite quail support influenza virus replication with receptors in their respiratory tract.^6^, ^7^ White leghorn specific pathogen‐free (SPF) chickens (*Gallus domesticus*) aged 6 weeks were also used. The number of birds and the sampling times differed between species due to the space limitations in our BSL3 facilities and not handling two different species on the same day. For each virus, five donor quail and five donor chickens were infected by the natural route with 10^6^ EID_50_ in 0.5 mL total volume phosphate‐buffered saline (0.1 mL intraocularly, 0.1 mL intranasally, 0.2 mL intraorally, and 0.1 mL intratracheally) as others have done.^8^ Twenty‐four hours after infection, donor birds were mixed with five direct‐contact birds of the same species in the same cage, five airborne‐contact quail were placed in an adjacent cage approximately 10 cm from the donor quail cage, and three airborne‐contact chickens were placed in an adjacent cage approximately 10 cm from the donor chicken cage. Allentown Caging 3‐tier Poultry Racks have doors made with vertical wire bars approximately 15 mm apart, so there is minimal barrier for airborne droplets between adjacent cages (cage dimensions 102 cm L × 61 cm W × 56 cm H). For quail, cloacal and oropharyngeal swabs were collected at 2, 4, 6, 8, and 10 days postinfection (dpi). On Day 10, only the donor quail were swabbed and only in the oropharyngeal cavity. For chickens, swabs were collected from contact birds on every other day and from the cloacal and oropharyngeal cavities at 2, 4, and 6 dpi. Swabs were collected from the cloacal and oropharyngeal cavities of donor chickens at 3, 5, and 7 dpi. The virus titers of swabs were determined in the allantoic cavities of 10‐day‐old embryonated chicken eggs, and the 50% egg infectious dose (EID_50_) was calculated by the method of Reed and Muench.^9^ For statistical analysis, the mean swab titers and SD error bars were calculated using GraphPad Prism version 7.03 for Windows (GraphPad Software, La Jolla, CA).

**Figure 1:**
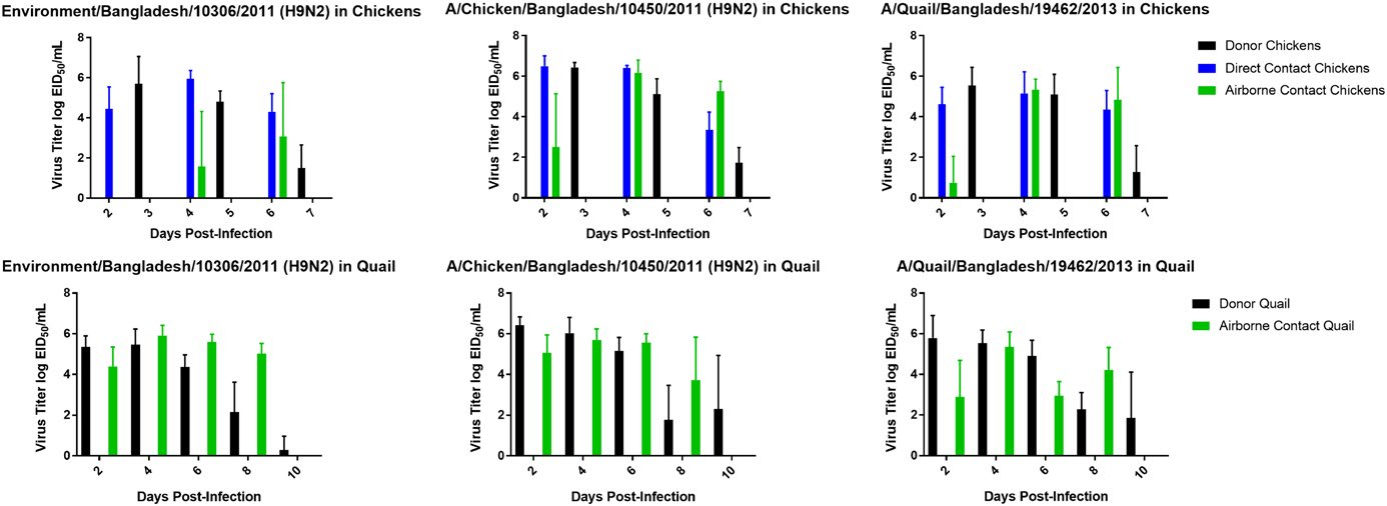
Transmission of H9N2 influenza viruses in chickens and quail. Chickens and New World quail were infected by the natural route with H9N2 influenza viruses as described in the text. The virus titers are given as the means and standard deviations for the samples from the birds in each group. Only the airborne‐infected quail were assayed because all five of the quail in separate cages were tested positive for virus by Day 2

Each of the H9N2 influenza viruses from chickens, quail, and the environment replicated to a high titer (EID_50_ 5.4‐6.4 log_10_/mL) in the upper respiratory tract of both chickens and quail (Table 1, Figure 1). Virus was also shed in the feces but with much lower titers (0.5‐2.7 log_10_/mL). In donor chickens, peak shedding of virus occurred at 3 dpi and the titers fell to background levels by Day 7. Similar virus titers were measured for each of the three viruses at 3, 5, and 7 dpi (data not shown). Shedding in quail donors peaked at 4 dpi, and shedding was sustained beyond 8 dpi in three of five donor birds for each virus. At least one donor quail in each group shed virus orally for 10 days.

**Table 1:** Replication and disease signs of H9N2 viruses in chickens and quail

H9N2 viruses	Oropharyngeal titer^a^	Cloacal titer^a^	Disease signs	Death	HA receptor specificity	Sequence differences		
						HA	NA	PB1‐F2
Chickens								
A/chicken/Bangladesh/10450/2011	6.4 ± 0.2	2.7 ± 1.7	None	None	α 2‐6	—	—	—
A/quail/Bangladesh/19462/2013	5.6 ± 0.9	1.9 ± 0.6	None	None	α 2‐6	6^d^	29	10
A/environment/Bangladesh/10306/2011	5.7 ± 1.3	0.8 ± 0.7	None	None	α 2‐6, α 2‐3	8	3	5
Quail								
A/chicken/Bangladesh/10450/2011	6.4 ± 0.4	1.7 ± 2.3	None	None				
A/quail/Bangladesh/19462/2013	5.8 ± 1.1	1.1 ± 0.6	++^b^	None				
A/environment/Bangladesh/10306/2011	5.4 ± 0.5	0.5 ± 0.7	None	++^c^				

a Mean titer in donor birds ± standard error (log_10_/mL) at 3 days postinfection.

b Lethargy in 4/5 donors and 5/5 direct contacts and conjunctivitis in 5/5 donors.

c Death in 1/5 donors, 1/5 direct contacts, and 2/5 airborne contacts.

d Amino acid differences between the environmental isolate and the other two isolates. The differences in other gene segments were less than those shown.

We determined the direct and airborne transmissibility of the H9N2 influenza viruses in chicken and quail, both between birds housed in the same cage and between birds in adjacent cages. Each of the viruses studied transmitted by direct contact and airborne contacts in chickens and by airborne contacts in quail (Figure 1). Direct transmission in quail was not evaluated because the airborne‐contact birds were already infected by the second day. In chickens, each virus had spread to direct‐contact birds by Day 2. In quail, H9N2 isolates had also transmitted to airborne‐contact birds in adjacent cages by Day 2 (Figure 1). In chickens, all three viruses transmitted to airborne contacts but varied in their rate and degree of transmission. A/Environment/Bangladesh/10306/2011 virus was not shed by airborne‐contact chicken until 4 dpi, and one of the three airborne‐contact chickens never shed the virus. In the other two groups of chicken airborne contacts, two of the three birds shed virus at 2 dpi and all of them shed virus on days 4 and 6 postinfection. In quail, airborne transmission occurred with all three viruses by Day 2, respiratory shedding of virus peaked by Day 4 (Figure 1), and shedding continued through Day 8.

None of the three H9N2 viruses studied caused overt disease signs in chickens, whereas the viruses differed in their effects on quail. Quail infected with the A/chicken/Bangladesh/10450/2011 (H9N2) virus showed no overt disease signs, whereas birds infected with the two isolates from quail (ie, the isolate from a bird and the environmental isolate from a quail cage) showed varying levels of disease signs (Table 1). A/quail/Bangladesh/19462/2013 (H9N2) caused lethargy in the donor and direct‐contact birds and conjunctivitis in the inoculated donor quail. This virus, which was isolated 2 years after the other two, had 29 amino acid substitutions in its neuraminidase and 10 amino acid substitutions in its PB1‐F2, as compared with the chicken isolate (Table 1). The A/environment/Bangladesh/10306/2011(H9N2) isolate from a quail cage caused sporadic mortality in donor, direct‐contact, and airborne‐contact birds. Surprisingly, the infected quail showed no overt signs of disease before death. According to amino acid sequences, the hemagglutinin protein of this virus possessed both α 2‐3 and α 2‐6 sialic acid binding properties, whereas that of the other isolates exhibited only α 2‐6 sialic acid receptor binding.

## 2 CONCLUSIONS

This study shows that the New World quail (Bobwhite species) is more susceptible to infection with Bangladeshi H9N2 influenza virus than chicken. Each of the H9N2 viruses replicated to a high titer in the respiratory tract of both chickens and quail, with low‐level shedding in the feces. The viruses all transmitted by direct contact and airborne transmission in chickens and by airborne transmission in quail, but the efficiency of airborne transmission to chickens varied. The differences in morbidity and mortality caused by the three H9N2 viruses in quail may be associated with sequence differences.

In this study, quail were more severely affected by the H9N2 viruses containing five gene segments from HPAI H7N3 than were chickens. H9N2 viruses from Pakistan containing four gene segments (PB2, PB1, PA, and NS) from HPAI H7N3‐affected quail more severely than chickens and caused sporadic deaths (one donor, one direct contact, and two airborne contacts).^10^ The Pakistani H9N2 virus caused more severe infections in chicken than did the Bangladeshi virus, probably because the Pakistani virus contained the PB2 gene segment from the HP H7N3, whereas the Bangladeshi viruses did not possess that gene segment.

Although fewer airborne‐contact chickens (3 per group) were used than in New World quail (5 per group), differences in airborne transmissibility are apparent and emphasize the risk factors of New World quail present in live bird markets: Variable transmission occurred in chickens among viruses tested, but high transmission occurred in New World quail. Quail (Old World) have been recognized as efficient spreaders of H9N2 influenza viruses and have been banned from live poultry markets in Hong Kong since 2002.^11^ This study alerts poultry farmers that New World quail are a potential threat at spreading influenza viruses. In Dhaka, Bangladesh, quail are sold in a separate wholesale market, but in the retail markets, quail and other minor poultry tend to remain in the markets longer than chickens, which are sold out daily.^12^ We do not know whether quail containing a gene constellation that differs from those found in birds in Hong Kong are the source of the H9N2 viruses in chickens in Bangladeshi live poultry markets. We do know that chickens in the Bangladeshi markets are more often infected than chickens sampled on the farms in that region, which suggests that poultry in the markets are the primary source of H9N2 viruses and that quail are a major contributor thereof.^12^

## References

[1] Guan Y , Shortridge KF , Krauss S , et al. Molecular characterization of H9N2 influenza viruses: were they the donors of the “internal” genes of H5N1 viruses in Hong Kong? Proc Natl Acad Sci USA. 1999;96:9363‐9367. 10430948 10.1073/pnas.96.16.9363 PMC17788

[2] Guo YJ , Krauss S , Senne DA , et al. Characterization of the pathogenicity of members of the newly established H9N2 influenza virus lineages in Asia. Virology. 2000;267:279‐288. 10662623 10.1006/viro.1999.0115

[3] Alexander DJ . Report on avian influenza in the Eastern Hemisphere during 1997‐2002. Avian Dis. 2003;47:792‐797. 14575066 10.1637/0005-2086-47.s3.792

[4] Iqbal M , Yaqub T , Mukhtar N , et al. Infectivity and transmissibility of H9N2 avian influenza virus in chickens and wild terrestrial birds. Vet Res. 2013;44:100. 24134616 10.1186/1297-9716-44-100 PMC4015117

[5] Shanmuganatham K , Feeroz MM , Jones‐Engel L , et al. Genesis of avian influenza H9N2 in Bangladesh. Emerg Microbes Infect. 2014;3:e88. 26038507 10.1038/emi.2014.84 PMC4317637

[6] Kimble B , Nieto GR , Perez DR . Characterization of influenza virus sialic acid receptors in minor poultry species. Virol J. 2010;7:365. 21143937 10.1186/1743-422X-7-365 PMC3022680

[7] Pantin‐Jackwood MJ , Miller PJ , Spackman E , et al. Role of poultry in the spread of novel H7N9 influenza virus in China. J Virol. 2014;88(10):5381‐5390. 24574407 10.1128/JVI.03689-13 PMC4019135

[8] Perez DR , Lim W , Seiler JP , et al. Role of quail in the interspecies transmission of H9 influenza A viruses: molecular changes on HA that correspond to adaptation from ducks to chickens. J Virol. 2003;77(5):3148‐3156. 12584339 10.1128/JVI.77.5.3148-3156.2003 PMC149770

[9] Reed LJ , Muench H . A simple method of estimating fifty percent endpoints. Am J Epidemiol. 1938;27:493‐497.

[10] Iqbal M , Yaqub T , Reddy K , et al. Novel genotypes of H9N2 influenza A viruses isolated from poultry in Pakistan containing NS genes similar to highly pathogenic H7N3 and H5N1 viruses. PLoS ONE. 2009;4:e5788. 19517011 10.1371/journal.pone.0005788 PMC2690689

[11] Lau EH , Leung YH , Zhang LJ , et al. Effect of interventions on influenza A (H9N2) isolation in Hong Kong's live poultry markets, 1999‐2005. Emerg Infect Dis. 2007;13:1340‐1347. 18252105 10.3201/eid1309.061549

[12] Turner JC , Feeroz MM , Hasan MK , et al. Insight into live bird markets of Bangladesh: an overview of the dynamics of transmission of H5N1 and H9N2 avian influenza viruses. Emerg Microbes Infect. 2017;6:e12. 28270655 10.1038/emi.2016.142 PMC5378921

